# Treatment of Boils

**Published:** 1891-08

**Authors:** 


TREATMENT OF BOILS.—Dr. Veiel gives in the *Gazette Medicale de Liege* the following process for destroying boils: Where the boil can be aborted, which is very seldom, he injects it with carbolic acid, perforates the furuncle with a silver needle, and introduces a crystal of sublimate. He attempts to prevent the formation of a core by applying hot cataplasms, and also applies a 1-10 per cent. solution of the sublimate. If this makes the skin very irritable, a 4 per cent. boric acid solution is applied instead. These applications are made during the day. At night an ointment of 6 drachms each of oxide of zinc and vaselin, and one-half drachm of boracic acid is used. He prevents the formation of new boils by applying a solution of the bi-chloride of mercury in 90 per cent. of alcohol three times a day.—*Journal de Medicine.*

